# The Rational Treatment of Epilepsy

**Published:** 1876-11

**Authors:** Reuben A. Vance

**Affiliations:** President of the Ohio Valley Medical Association. Gallipolis, Ohio


BUFFALO

### Medical and Surgical Journal.

VOL XVI. NOVEMBER, 1876. No. 4.

### Original Communications.

:o:

ART. I.—*The Rational Treatment of Epilepsy. A Brief Review of the Phenomena and Pathology of that Disease, with observations relative to the value of the Ophthalmoscope as a means of determining the varying states of the Intra-Cranial Circulation.* By REUBEN A. VANCE, M. D., President of the Ohio Valley Medical Association. Gallipolis, Ohio, Nov. 1876.

It is a matter of common observation, that cases of Epilepsy are affected very differently by the same method of treatment; that the same drug administered in the same quantities to cases that apparently resemble each other in every particular, will at times act in a diametrically opposite manner. The bromide of potassium, for instance, will at once relieve certain cases, while in certain others it invariably increases the number and severity of the paroxysms.

As a general rule, it can be said that the cases of epilepsy benefitted by the bromide will be those characterized by the diurnal occurrence of the fits, while those injured by it, will be the cases in which the paroxysms are either exclusively nocturnal, or occur principally by night.

It is also a matter of experience that those cases in which the fits are controlled by the bromide, present something like the following history: For a variable period after commencing with this drug the paroxysms cease, but sooner or later, in the vast majority of cases, they return in an altered form and recur with increased frequency. However much the dose of the bromide is now increased, it will fail to control the paroxysms, and it will be necessary to stop its administration for several weeks. As soon as the system recovers its natural tone, the bromide can be recommenced, and another interval of freedom will ensue to be followed, in turn, by a similar series of convulsions.

I claim that the difference in the character of the convulsions, as well as the different effects of this remedy, are due to variations in the cerebral circulation; that in one class of cases—those occurring principally by day—there exists, as the efficient cause of the convulsive seizures, a congested condition of the brain; while in those which recur either generally or exclusively by night, there is present the opposite state, an anaemic condition of the cerebral organs. The physiological effect of the bromide of potassium is to diminish the amount of blood circulating through the brain. This fact explains why this drug relieves those cases in which the disease depends upon cerebral hyperæmia and aggravates those in which the efficient cause is an anæmic condition of the brain.

I also declare that the ophthalmoscope affords a ready means of determining the state of the cerebral circulation, thus indicating the treatment to be pursued in a given case. The vascularity of the optic disk and retina is indicative of the blood-supply of the brain. Hyperæmia in the one indicates hyperæmia in the other, and *vice versa*. Treatment is always to be directed to the correction of any abnormality in the intra-cranial circulation, and for this purpose remedies are to be used which experience and observation have demonstrated to possess the power of producing the desired change; for instance, bromide of potassium or oxide of zinc, when it is desirable to relieve a hyperæmic state, and belladonna or strychnia, when we wish to counteract an anæmic condition.

While these remedies, judiciously administered, always tend to relieve the patient, such relief is generally but temporary in character. This fact is explained as follows: In those widely opposite conditions, cerebral hyperæmia and anæmia, we have the efficient causes of these two classes of epileptiform symptoms, and in the physiological effects of remedies like the bromide of potassium and sulphate of strychnia, we have a rational explanation of the manner in which they can be relieved. Were these drugs so endowed as to cease their effects when they have restored the cerebral circulation to a normal standard, the problem of the cure of the vast majority of epileptics would be solved at once. Unfortunately, however, such is not the case; these remedies tend to produce but one effect, and that, if carried too far, will result in the production of a condition equally competent to cause epileptiform symptoms. For instance, the condition of cerebral hyperæmia causing convulsions may be so affected by medicines as to gradually pass away, and be succeeded by a normal state of the intra-cranial circulation. With the continued administration of the same remedy, the amount of blood will diminish more and more until an anæmic condition is induced. A similar result follows the administration of remedies calculated to increase the amount of blood in a brain where the epileptiform symptoms are due to cerebral anæmia. With the increased quantity of blood sent into that organ, a normal condition is produced; as the action of the drug continues, this is succeeded by hyperæmia. In other words, any remedy which has the power to affect the cerebral circulation, may, when pushed too far, result in the production of a condition competent to again excite convulsions.

If such diametrically opposite cerebral conditions can cause epileptiform symptoms, and if the methods of treatment which relieve them temporarily are liable from their own effects, to bring them on again, how are we to regulate our remedies so as to prevent these bad results? Have we any means of observing the changes in the brain circulation which will enable us to watch the effects of our medicines, and so avoid the production of these extreme conditions? I claim that we have, in the ophthalmoscope, an instrument which will enable us to accomplish the desired object. I have reported a number of cases in which a successful result has been obtained by treatment directed in accordance with the indications afforded by opthalmoscopic examinations. These examinations should be repeated as often as twice a week during the time the patient is under treatment. No case can be considered cured until an interval of one year has elapsed from the last convulsion, but the number of patients who have been radically cured by this course of procedure is now so large that the therapeutic measures by which they regained their health deserve to be ranked among the most valuable resources at the command of the physician.

---

For a more complete exposition of the principles advanced in the above, the reader is referred to the *New York Medical Journal*, February, 1871, and the *American Journal of Syphilography and Dermatology*, July, 1871.

